# Tele‐Genetic Versus Onsite Counseling for APOE Disclosure: A 6‐Month Study of Empowerment, Psychological Distress, and Behavioral Outcomes in Cognitively Diverse Individuals

**DOI:** 10.1002/jgm.70041

**Published:** 2025-10-01

**Authors:** Marina Makri, Ioanna Antigoni Angelidou, Despoina Moraitou, Liana Fidani, Thomas Tegos, Vasileios Kimiskidis, Magdalini Tsolaki

**Affiliations:** ^1^ Department of Neurology, School of Medicine, Faculty of Health Sciences Aristotle University of Thessaloniki Thessaloniki Greece; ^2^ Greek Association of Alzheimer's Disease and Related Disorders Thessaloniki Greece; ^3^ Laboratory of Neurodegenerative Disease, Center for Interdisciplinary Research and Innovation (CIRI—AUTh), Balkan Center Aristotle University of Thessaloniki Thessaloniki Greece; ^4^ Network Aging Research (NAR) Heidelberg University Heidelberg Germany; ^5^ Department of Cognition, Brain and Behavior, School of Psychology Aristotle University of Thessaloniki Thessaloniki Greece; ^6^ Department of Medical Biology–Genetics, School of Medicine, Faculty of Health Sciences Aristotle University of Thessaloniki Thessaloniki Greece

**Keywords:** Alzheimer's disease, APOE, genetic counseling, genetic testing, health‐behavior modification, psychological reaction

## Abstract

**Background:**

With the increasing demand for clinical genetic services and the lack of clinical geneticists, tele‐genetics is utilized in clinical genetics to improve cost efficiency and equitable access to health services. But is tele‐genetic counseling (GC) for APOE genotype disclosure for Alzheimer's disease (ad) risk as effective as onsite interventions?

This study examines behavioral, psychological, empowerment, and risk recall responses following the disclosure of genetic results of AD risk to first‐degree relatives of people with AD (PwAD) in Greece.

**Materials and Methods:**

Participants (*N* = 93) were randomly assigned to one of two GC interventions. Additional grouping variables included the cognitive status (healthy relatives of PwAD or people with mild cognitive impairment [MCI]) and the genetic test result (ε4‐positive or not). Throughout the three time points (baseline/before GC, 3 and 6 months post‐disclosure), participants completed questionnaires for psychological well‐being, empowerment, risk recall, and behavioral adaptations. Repeated‐measures ANOVA, Mann–Whitney *U* tests, logistic regression, Friedman test, and *χ* ^2^ tests were used to examine changes in different scores.

**Results:**

Ninety‐three adults (mean age 64.77 years, years of education 13.72, 61% female) were randomly assigned to online (46 participants) and onsite (47 participants) groups. There were no statistically significant differences between the two intervention groups in psychological and empowerment scores. However, people with MCI have substantially lower odds of recalling risk after 6 months and lower empowerment scores. People with ε4 were much more likely to endorse behavior change and indicated higher distress scores.

**Conclusion:**

Tele‐GC can serve as an effective intervention for APOE disclosure for healthy relatives of PwAD and people with MCI.

## Graphical abstract

This study examines behavioral, psychological, empowerment, and risk recall responses following the disclosure for genetic results of Alzheimer's disease risk (online and on‐site) to first‐degree relatives of people with AD (healthy relatives or with mild cognitive impairment‐MCI) in Greece. There were no differences between the two intervention groups. MCI participants have lower odds of recalling risk and empowerment scores. People with ε4 were more likely to endorse behavior change and higher distress scores.

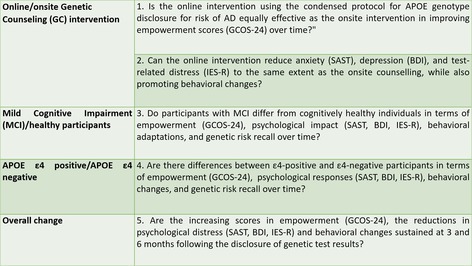

## 1 Introduction

Over the last decade, the use of telemedicine has significantly increased across various sectors of healthcare worldwide, primarily to enhance cost‐effectiveness and provide equitable access to medical services in remote areas or for individuals with mobility challenges. “In this approach, treatments, diagnoses, therapeutic options, and follow‐up recommendations are determined using patient data, documentation, and other information transmitted via telecommunications systems” [1]. As the demand for clinical genetic services rises, paired with a shortage of clinical geneticists and genetic counselors, established genetic centers can leverage telemedicine, a practice known as “tele‐genetics” [2, 3, 4]. This can serve as an invaluable method for genetic counseling (GC), utilizing telephone or video formats, i.e., digitally or remotely, and allows professionals the opportunity to share their expertise [5]. The COVID‐19 pandemic has further accelerated the development and acceptance of tele‐genetics to minimize in‐person appointments between patients and healthcare professionals [3, 4]. According to a recent survey by the National Society of Genetic Counselors, tele‐genetics (audiovisual) emerged as the leading service delivery model in North America in 2021, utilized by 82% of respondents, closely followed by face‐to‐face GC at 81% and telephone sessions at 74% [6].

GC is a communication process designed to help individuals address the presence or potential risk of a genetic disorder within their family [7]. It supports not only those affected by a genetic condition but also their family members in understanding and coping with the implications of having an inherited disease in the family [7]. Reliable measurement tools are used to assess the effectiveness of GC interventions, including scales for depression, anxiety, or distress, as well as instruments to measure empowerment, all aimed at continuously improving the services. Empowerment is defined as “a set of beliefs that allow individuals from a family impacted by a genetic condition to feel that they have some control over and hope for the future” [8]. To our knowledge, this is the first study to measure the effects on participants' empowerment after tele‐GC appointments compared to face‐to‐face GC.

Genomic medicine provides options, such as genetic tests, which can estimate an individual's likelihood of developing specific diseases in the future [9]. Concerns have been raised about the significance of one particular test: the ε4 allele of the apolipoprotein E (APOE) gene [10, 11]. The presence of the ε4 allele increases an individual's risk for the common, late‐onset form of Alzheimer's disease (ad). While 10% to 15% of the general population will develop AD by age 85, those with one copy of the APOE ε4 gene face a risk of 25% to 40%, and those with two copies face a risk of 40% to 55% [12]. Major professional organizations have issued recommendations against conducting APOE testing for AD risk [10, 13]. Their concerns stem from the test's inability to identify individuals who will develop the disease definitively, given the influence of environmental factors and the complex interplay with other genes. Additionally, there is a risk of causing psychological distress, as there are currently no validated medical solutions available to mitigate or prevent the disease [11]. Nevertheless, even without such interventions, some individuals find personal value in the test, as it enables them to prepare for the future, adopt risk‐reduction behavioral changes, and participate in clinical trials of disease‐modifying therapies for AD [14, 15, 16].

Given the strong public interest in genetic susceptibility testing regarding ad and the national priorities on the prevention and early detection of ad, it is likely that the number of individuals with mild cognitive impairment (MCI) and healthy individuals seeking APOE‐based risk information about ad will continue to rise [11, 17]. The largest research study on disclosing the APOE genotype for the risk of ad was the Risk Evaluation and Education for Alzheimer's Disease (REVEAL) study [13, 14]. This study primarily focused on informing healthy individuals about their risk of ad and concluded that disclosing APOE status resulted in only temporary psychological distress for those found to have inherited ε4 alleles. However, the participants included in the studies were generally many years away from displaying any symptoms, even if they were to develop ad. There remain concerns about the impact of disclosing genetic risk to individuals currently experiencing memory issues who may be approaching the onset of ad dementia. Conversely, many studies now recommend adhering to specific GC protocols, which are both effective and safe, for disclosing APOE genotype related to the risk of ad [13, 18, 19, 20]. Roberts et al. (2012) and Green et al. (2015) demonstrated the safety of a brief disclosure protocol for announcing APOE genotype in relation to ad risk, which required less clinician time than the extended protocol and followed highly condensed pretest education and counseling [18, 21].

Despite the identification of more genetic diseases, growing complexity, and heightened awareness of genetic testing, access to GC remains limited globally, including in Greece, often falling short of needs due to a shortage of trained professionals such as genetic counselors and geneticists [22]. In Greece, genetic services are currently provided by physicians working alongside clinical laboratory geneticists because there is a lack of medical geneticists and genetic counselors. The availability of GC services in Greece is notably scarce, as only six out of 52 clinics and hospitals with cancer units in four cities offer GC services [23]. Consequently, individuals with inherited disorders and their families are unable to fully benefit from genetic testing, GC, management, and monitoring, which could improve their quality of life. A potential solution to these obstacles is tele‐GC. Until the advent of the COVID‐19 pandemic, comprehensive legislative initiatives on telemedicine were lacking in Greece, which posed a significant barrier to its widespread use. Following the introduction of a new legislative provision, medical procedures can now be explicitly carried out remotely through the telemedicine system using digital applications [24]; [25]. The use of tele‐genetics has not previously been studied in the Greek context, and this study aimed to examine psychological and behavioral responses following the online and onsite disclosure of genetic results of AD risk to first‐degree relatives of people with AD who are healthy and with MCI in Greece. The primary aim was to evaluate the impact of tele‐GC and face‐to‐face GC on participants' empowerment, psychological well‐being (anxiety, depression, and distress symptoms), behavioral adaptations, and risk recall over time. Results from this investigation may provide relevant information to stakeholders in order to truly enhance individual psychological well‐being, empowerment, and lifestyle changes toward genetic risk information management by using more of the tele‐genetic approach.

We hypothesized that both types of interventions would be effective and that participants in online sites would experience no greater anxiety, depression, or distress than participants in onsite interventions, as well as similar behavioral adaptations and risk recall over time (Research Questions 1 and 2). We expect these scores to remain over time (Research Question 5). Furthermore, we hypothesized that participants with MCI would not differ from cognitively healthy individuals in terms of psychological impact, behavioral adaptations, and genetic risk recall (Research Question 3). Finally, we hypothesized that participants who learned they were APOE ε4–positive would experience no greater psychological and behavioral impact and risk recall than participants who learned they were APOE ε4–negative (Research Question 4). All research questions and their underlying hypotheses are illustrated in Table 1.

**TABLE 1:** Research questions of the study.

Online/onsite GC intervention	1 Is the online intervention using the condensed protocol for APOE genotype disclosure for risk of ad equally effective as the onsite intervention in improving empowerment scores (GCOS‐24) over time?
	2 Can the online intervention reduce anxiety (SAST), depression (BDI), and test‐related distress (IES‐R) to the same extent as the onsite counseling, while also promoting behavioral changes?
MCI/healthy participants	3 Do participants with MCI differ from cognitively healthy individuals in terms of empowerment (GCOS‐24), psychological impact (SAST, BDI, IES‐R), behavioral adaptations, and genetic risk recall over time?
APOΕ ε4–positive/APOΕ ε4–negative	4 Are there differences between ε4‐positive and ε4‐negative participants in terms of empowerment (GCOS‐24), psychological responses (SAST, BDI, IES‐R), behavioral changes, and genetic risk recall over time?
Overall change	5 Are the increasing scores in empowerment (GCOS‐24), the reductions in psychological distress (SAST, BDI, IES‐R), and behavioral changes sustained at 3 and 6 months following the disclosure of genetic test results?

## 2 Methods

### 2.1 Study Design

This study is an experimental pilot study, where participants were assigned to two different GC interventions (online and onsite). The study follows a longitudinal design with three measurement points, at the baseline T0 (prior to genetic testing and counseling), T1 (3 months post‐disclosure), and T2 (6 months post‐disclosure).

Participants were randomly allocated into two groups: an online and an onsite group. The online group participated in all sessions through videoconferencing, and the onsite participants had the first two sessions in a face‐to‐face format. First, matching and then randomization were applied to assign cases to groups. Matching procedures reduce baseline differences between intervention and control groups, and randomization can prevent bias in assigning participants to groups [26]. Groups of two recruited cases were matched as closely as possible for age, gender, education, and level of anxiety and depression (stratified randomization). Before the inclusion of participants, an information technology technician performed randomization using an algorithm. All of the independent evaluators were blind to group assignment, and the participants were not informed of primary outcome measures or the study hypothesis.

### 2.2 Study Population

Greek adults with at least one living or deceased first‐degree relative affected by late‐onset ad (onset ≥ 60 years) were eligible for participation. We recruited individuals with no cognitive decline and with amnestic MCI. The diagnosis of MCI was according to the Petersen criteria, following a neuropsychological assessment (adequate general cognitive function with Mini‐Mental State Examination score ≥ 24) and a neurological examination [27, 28].

To ensure MCI participants' safety, we required participants to enroll and attend sessions with a companion. This instruction was mandatory for all MCI participants in order to have someone accompany them to the counselor's office or better explain the issues of the procedure. A companion was not required for cognitively healthy participants.

There were no age criteria. Exclusion criteria included severe anxiety or depression as judged by a clinician and as indicated by scores on mood scales.

Study participants were reached through the 1st Department of Neurology of Aristotle University of Thessaloniki (Greece) and the Greek Association of Alzheimer's Disease and Related Disorders (GAADRD). The current study sample was recruited between September 2023 and March 2024. To calculate the sample size, a priori power analysis was obtained using G*Power 3.1 to determine parameters, a medium effect size (*d* = 0.25), and an *α* of 0.05. Results showed that a total sample of 56 participants was required to achieve a power of 0.80 [29].

### 2.3 Data Collection Procedure

**FIGURE 1:**
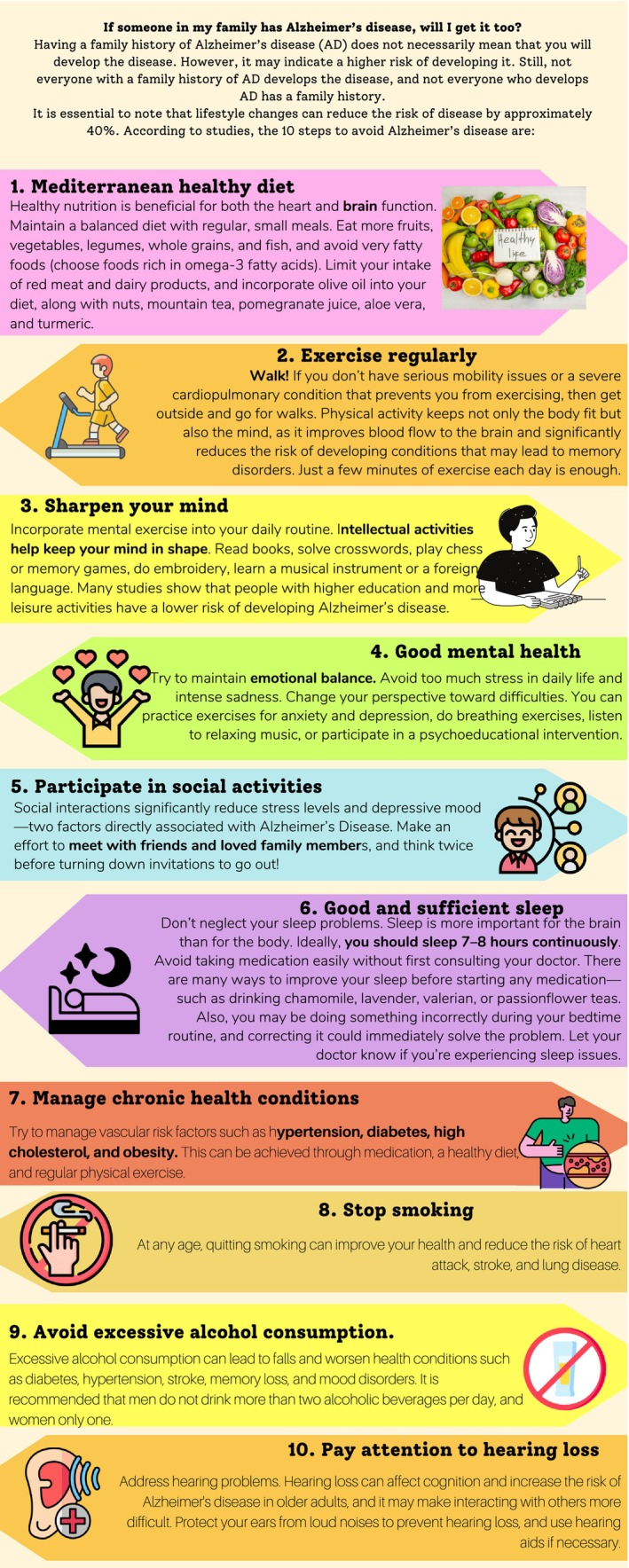
Brochure in English.

This study followed the condensed protocol for GC, which has been proven to be a safe protocol for APOE disclosure [18]. The following steps were followed:

1. Potential participants were initially contacted via telephone to determine eligibility, as well as to obtain verbal informed consent and demographic information. Eligible participants were then mailed an educational brochure, which included information about AD, the APOE gene and genotypes, and other risk factors associated with AD, as well as information about the study.
2. A first session, which lasted about 30 min, was subsequently scheduled and conducted, during which participants had the opportunity to meet a genetic counselor and ask questions about the educational brochure. A written informed consent was obtained, and baseline measures for measuring the cognitive decline, empowerment, anxiety, and depression symptoms were administered. The family history was discussed, and the genetic counselor created a family pedigree. It is crucial to highlight that, unlike in other countries, Greece has no national regulation for the profession of genetic counselor. Genetic counselors are not required to have certification to practice. In our study, biologists conducted the sessions.
3. A blood draw for genetic testing was done, and samples were sent to a diagnostics laboratory for genotyping. A genetic counselor performed disclosure of the APOE test results during the second appointment, approximately 2 months after the blood draw. During this session, which lasted 20 min on average, the genetic counselor verbally disclosed participants' results using a scripted template, emphasizing participants' genotypes and the number of risk‐increasing alleles. Participants were also given written materials that included a summary statement containing their APOE genotype and a leaflet with risk factors for AD (Figure 1), their cumulative impact, and risk‐modifying behaviors. It is crucial to highlight that, in this session, it was underlined that the risk of AD has not been proven to affect disease progression. This leaflet followed the Finger study measures [30]. A presentation and a discussion on these measures were followed. This discussion emphasized that although the APOE ε4 allele is an important risk factor for AD, it is neither necessary nor sufficient to cause the disease and also emphasized that following the modifying behaviors does not guarantee they can prevent the disease. Near the end of the disclosure session, the genetic counselor confirmed the participant's understanding of the information, corrected any misunderstandings, and discussed their emotions.
4. Participants were followed for 3 and 6 months after disclosure sessions, with assessments conducted online and via telephone. A 1‐year follow‐up appointment was originally planned but was shortened to 6 months to reduce demands on participants and because prior studies [13, 21] and also anecdotal descriptions had suggested that there were no additional changes in psychosocial outcomes after 6 months [11]. In these follow‐up appointments, validated psychological scales, a risk recall survey, and a questionnaire about behavioral changes were administered.

### 2.4 Survey Measures

#### 2.4.1 Demographics

Self‐reported gender, age, and education status were elicited during the initial phone interview. Education was reported as the number of years of schooling the participant completed.

#### 2.4.2 Family History

Participants self‐reported the total number of relatives they had with diagnosed ad or undiagnosed progressive dementia syndrome. This information was used to create a metric variable representing the number of affected family members.

#### 2.4.3 The Empowerment of the GC Intervention

The Greek version of the Genetic Counseling Outcome Scale (GCOS‐19) was included to capture the effects of the two types of GC by measuring changes in participants' empowerment levels before GC (baseline) compared to after the intervention (3 months after the appointment of genetic disclosure, T1). The Greek GCOS contains 19 questions regarding their experience of GC, and responses are given on a 7‐point Likert scale, ranging from 1 = strongly disagree to 7 = strongly agree [31]. Scoring is done by assigning 1 to 7 points to each answer, and the final score can range from 19 to 133 points. A mean increase of more than 10.3 points, on a group level, indicates that a minimal clinically important difference (MCID) has been achieved, thus providing clinical utility and interpretability to the scores [32].

#### 2.4.4 Cognitive and Psychometrical Instruments

##### 2.4.4.1 Cognition

The Montreal Cognitive Assessment is a simple, self‐administered mental screening tool with high sensitivity in detecting mild mental disorders [33]. Its subtests focus on short‐term recall, visuospatial abilities, executive function, attention, concentration, working memory, language, and orientation in time and space. The test's total score is 30, which indicates intact cognitive function, and the cutoff scores range between 16 and 26 points. It has been validated in the Greek population [34]. The scale was administered (baseline) to participants of this study to confirm the diagnosis of MCI and healthy participants.

##### 2.4.4.2 Anxiety

The Short Anxiety Screening Test (SAST‐10) version was validated and translated into Greek and is considered a reliable screening tool in primary healthcare [35]. It includes a 10‐item Likert‐type response scale and was first developed by Sinoff and colleagues for geriatric patients [36]. Participants completed the scale at three time points: in the first appointment (baseline T0) and 3 (T1) and 6 months (T2) after the appointment of genetic disclosure.

##### 2.4.4.3 Depression

The Beck Depression Inventory (BDI‐II) is widely used to evaluate depressive symptomatology in clinical and non‐clinical populations. It includes 21 items (scored from 0 to 3), and the scale's total score comes from their sum [37]. The Greek version of the BDI II showed very good psychometric properties, and thus, it can be administered in a Greek population, giving valid and reliable data. In our study, the administration of this scale followed the exact times of SAST‐10 (T0 → T2) [38].

##### 2.4.4.4 Distress

Distress was measured at 3‐ and 6‐month follow‐ups (T1 → T2) using the validated Impact of Event Scale—Revised (IES‐R) [39]. The IES‐R comprises 22 items in three subscales (eight intrusion items, eight avoidance items, and six hyperarousal items). Respondents are asked to rate each item on a scale of 0 to 4 (0 = not at all, 1 = a little bit, 2 = moderately, 3 = quite a bit, and 4 = extremely) according to the past 7 days. The total score on the IES‐R ranges between 0 and 88 [39]. To measure the distress after the genetic results in our sample, the Greek version of the IES‐R‐Gr was used [40].

##### 2.4.4.5 Recall of APOE Genotype

To assess participants' recall of their APOE genotype, they were asked the following item at 3‐ and 6‐month follow‐ups: “What were your APOE genetic test results?” Response options were ε2/ε2, ε2/ε3, ε2/ε4, ε3/ε3, ε3/ε4, ε4/ε4, and “do not remember.” The responses of participants who recalled the correct genotype were coded as “correct,” while the responses of those who recalled the incorrect genotype, who could not remember their genotype, or who did not answer the question, were coded as “not correct” [41]. We decided to include only the APOE genotype information and no other genetic information, as studies have shown that more complex information can overload the participants and cause a poor risk recall [41, 42].

##### 2.4.4.6 Measurement of Health‐Behavior Modification

We examined whether the APOE genotype of our participants and the GC intervention altered health behaviors. They were asked a question with possible multiple‐choice answers in the two follow‐ups. It is related to health behavior changes made for the purpose of ad prevention (As a result of your genetic test results, have you made any changes to your lifestyle?). They could answer the question about behavioral changes by marking different categories like “changes in diet, physical exercise, cognitive exercise, trying to have better psychology (e.g., no anxiety, depression), being more social, changing my sleep, changing my medication, and vitamins.”

### 2.5 Ethical Considerations

The study protocol was accepted by the Research Ethics Committee of the School of Medicine of Aristotle University of Thessaloniki (Committee Approved Meeting Number: 1/19.10.2021) and also by the Scientific and Ethics Committee of the Greek Association of Alzheimer's Disease and Related Disorders (Committee Approved Meeting Number: 91/07‐09‐2023). The study protocol development followed the ethical standards outlined in the Declaration of Helsinki. The Greek Law of Data Protection was respected through the anonymity and confidentiality of the data and according to the General Data Protection Regulation (EU) 2016/679 of the European Parliament. All participants took part voluntarily in the study. Informed consent was provided before the beginning of the survey, which included details on the study's main goals.

### 2.6 Statistical Analyses

Statistical analysis was performed using the IBM Statistical Package for Social Sciences (SPSS) Version 29.0.1.1. Initially, all data were collated into an SPSS file, where they were anonymized. After checking the file for missing data, 93 participants out of the total number (106) were included in the statistical analysis. Descriptive data analysis of sociodemographic characteristics was described by calculating distributions, mean scores, and standard deviations. Each research question in Table 1 was tested using appropriate statistical analyses based on the independent variable and outcome measure.

#### 2.6.1 Preliminary Data Checks

The normality of the data was assessed for each variable across hypotheses using a combination of visual inspection (histograms, Q‐Q plots) and statistical measures of skewness and kurtosis, with acceptable ranges typically falling between −2 and 2 [43]. Formal normality tests (e.g., Shapiro–Wilk, Kolmogorov–Smirnov) were not emphasized, as their sensitivity increases with sample size and minor deviations from normality can lead to statistically significant results even when the data are approximately normal [44, 45]. To gain an overview of the data and identify potential confounding variables, Spearman's correlation coefficient (*ρ*) was used for exploratory analysis of the dataset [46]. Any variable that showed a significant correlation with the outcome variable but was not central to the hypothesis was included as a covariate in the statistical analyses to control for its potential influence. The variables included in the correlation matrix were demographics like age, gender, education status, and family history of dementia (as a metric variable), as well as the intervention format (online vs. onsite), the diagnostic group (MCI vs. healthy controls), and the APOE ε4 carrier status (ε4+ vs. ε4−).

#### 2.6.2 Statistical Group Comparisons

The statistical approach varied depending on the research question, the dependent variable (GCOS‐24, IES‐R, SAST, BDI), and whether a covariate needed to be controlled. To evaluate the effectiveness of the online vs. onsite intervention, a mixed ANCOVA was conducted to assess changes in empowerment scores (GCOS‐24) over time, with time (T0 → T1) as a within‐subjects factor, group (online vs. onsite) as a between‐subjects factor, and diagnosis (MCI vs. healthy) and gene status (APOE ε4+ vs. APOE ε4−) as covariates. The reported effect size was partial *η* ^2^ for ANOVA and ANCOVA and Cohen *d* for *t* tests [47]. Differences between the online and onsite group in psychological distress were analyzed separately for SAST, BDI, and IES‐R: Due to substantial violations of normality assumptions identified by visual inspection (numerous outliers), skewness exceeding ±2, and significant results from the Kolmogorov–Smirnov and Shapiro–Wilk tests (*p* < 0.05), nonparametric tests were consistently applied for analyses involving SAST and BDI at all measurement points (baseline, 3 months, 6 months, T0 → T3). Specifically, Friedman test was used for within‐group comparisons over time, and the Mann–Whitney *U* test for between‐group comparisons at individual time points. In contrast, IES‐R, assessed at 3 and 6 months (T1 → T2), was analyzed using a mixed ANCOVA, adjusting for gene status (APOE ε4+ vs. APOE ε4−) as a covariate. To examine whether the online intervention promotes behavioral adaptations as effectively as the onsite intervention, behavioral responses at 3 and 6 months were converted into binary variables (yes = 1, no = 0) and analyzed using *χ* ^2^ tests. If a significant group difference was found, logistic regression was conducted to explore predictors of behavioral changes. For Research Questions 3 and 4, comparing MCI vs. healthy participants and APOE ε4+ vs. APOE ε4− individuals on GCOS‐24, psychological impact (SAST, BDI, IES‐R), behavioral adaptations, and genetic risk recall, the same statistical strategy was applied. ANCOVA models were used to control for relevant covariates when assumptions were met, while nonparametric analyses (Mann–Whitney *U*, Friedman test) were consistently employed for SAST and BDI due to normality violations. Risk recall was assessed at T1 and T2 and was assessed as binary variables (yes = 1, no = 0). If a significant group difference was found, logistic regression was conducted to explore predictors of behavioral changes and risk recall. For the fifth research question, to assess overall changes in empowerment (GCOS‐24), psychological distress (SAST, BDI, IES‐R), and behavioral adaptations over time, paired *t* tests were used for GCOS‐24 (T0 → T1) and IES‐R (T1 → T2), while the nonparametric Friedman test was used for SAST and BDI. McNemar's test analyzed behavioral changes over time.

## 3 Results

### 3.1 Sociodemographic and Baseline Characteristics

**FIGURE 2:**
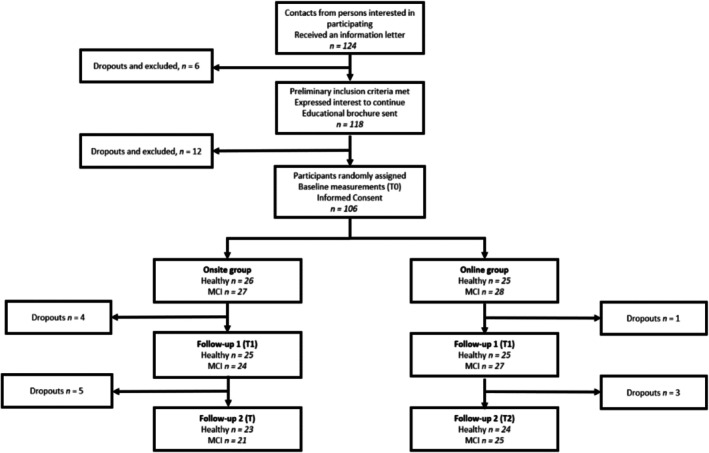
Diagram of the study.

In total, 102 people responded, and the final sample was composed of 93 participants, as nine participants did not complete all follow‐ups. Figure 2 presents the study flow diagram. Most of the participants were female (61,3%), with a mean age of 64.77 years and a wide range in age from 37 to 83 years. They were highly educated, with a mean of 13.72 years and a range of 6 to 24 years. 53.8% of the participants had MCI (50 people), and in total, 50.5% of participants took part in the online intervention (47 people). Regarding the gene status, 51.6% of the participants were APOE ε4–negative (48 people). Table 2 describes the demographic characteristics of the participants and the distribution of participants by mode of GC and diagnosis or genetic results. Most participants (83.9%) reported between one and three family members with dementia symptoms, with two relatives being the most common response (32.3%). As this variable was not identified as a confounder for the research questions, it was not included in further statistical analyses.

**TABLE 2:** Baseline characteristics of the 93 participants who attended GC sessions.

Characteristics	Full sample (*N* = 93)	%	Distribution of participants by mode of GC
**Age**			
Mean (SD)	64.77 (10.835)		65.19 (11.885) Onsite 64.35 (9760) Online
**Gender**			
Male	35	37.6	18 (51.4%) Onsite 17 (48.6%) Online
Female	58	62	29 (50.0%) Onsite 29 (50.0%) Online
Diverse	0	0	0
**Education**			
Mean (SD)	13.72 (3.809)		13.30 (3.375) Onsite 14.15 (4.200) Online
0–6 years (primary education)	9	9.7	4 (44.4%) Onsite 5 (55.6%) Online
7–12 years (secondary education)	24	25.8	16 (66.7%) Onsite 8 (33.3%) Online
>12 years (tertiary education)	60	64.5	27 (45.0%) Onsite 33 (55.0%) Online
**Cognitive status**			
MCI	50	53.8	26 (52.0%) Onsite 24 (48.0%) Online
Healthy	43	46.2	21 (48.8%) Onsite 22 (51.2%) Online
**Gene status (genetic risk result)**			
APOE ε4–positive	48	51.6	27 (56.3%) Onsite 21 (43.8%) Online
APOE ε4–negative	45	48.4	20 (44.4%) Onsite 25 (55.6%) Online
**Intervention group**			
Online	46	49.5	46 (49.5%)
Onsite	47	50.5	47 (50.5%)

### 3.2 Online Compared to Onsite Intervention

A mixed ANCOVA was conducted to examine whether the online intervention was equally effective as the onsite intervention in improving empowerment scores (GCOS‐24) over time. Diagnosis (MCI vs. cognitively healthy) and APOE ε4 status (ε4‐positive vs. ε4‐negative) were included as covariates, as they correlated significantly with the scores of the GCOS‐24. Analysis shows that the GCOS‐24 scores increased significantly over time across all participants, regardless of group assignment (*F*[1, 89] = 21.578, *p* < 0.001, partial *η* ^2^ = 0.195). However, the time × group interaction was not significant (*F*[1, 89] = 1.499, *p* = 0.224, partial *η* ^2^ = 0.017), indicating that the rate of improvement did not differ between the online and onsite groups. Additionally, there was no significant main effect of the group at any time point (*F*[1, 89] = 0.475, *p* = 0.387, partial *η* ^2^ = 0.008), suggesting that intervention type had no meaningful impact on GCOS‐24 scores. The intervention type explained less than 1% of the variance, confirming that both groups benefited equally from the intervention. Only the covariate diagnosis is significant in this analysis and explains 47% of the variance of GCOS‐24 (*F*[1, 89] = 78.886, *p* = 0.001, partial *η* ^2^ = 0.470).

Repeated‐measures ANOVAs were planned to examine whether intervention type (online vs. onsite) influenced changes in psychological distress (BDI, SAST) over time. Due to a violation of sphericity and normality in BDI at T0 (kurtosis over 3) in the online group, nonparametric tests were used to analyze changes in BDI scores over time and between the intervention groups. A Friedman test indicated no significant change in BDI scores over time, *χ* ^2^(2) = 3.644, *p* = 0.162, suggesting stability across time points. Similarly, Mann–Whitney *U* tests showed no significant differences between the online and onsite groups at any time point (BDI (baseline): *U* = 847.00, *Z* = −1.812, *p* = 0.070; BDI (T1): *U* = 958.00, *Z* = −0.954, *p* = 0.340; BDI (T2): *U* = 952.50, *Z* = −1.002, *p* = 0.316). A similar pattern was observed for SAST scores, where normality was violated on several occasions. A Friedman test indicated no significant change in SAST scores over time, *χ* ^2^(2) = 4.414, *p* = 0.110, while the Mann–Whitney *U* tests showed no significant differences between the online and onsite groups at any time point (SAST [baseline]: *U* = 1050.00, *p* = 0.809; SAST [T1]: *U* = 962.50, *p* = 0.360; SAST [T2]: *U* = 875.50, *p* = 0.110). These results indicate that BDI and SAST scores remained stable over time and did not differ between intervention groups.

A mixed ANCOVA showed that IES‐R scores significantly decreased from T1 to T2 with a large effect across all participants (*F*[1,90] = 32.338, *p* < 0.001, partial *η* ^2^ = 0.264). But the rate of improvement did not differ between the online and onsite groups *F*[1,90] = 0.201, *p* < 0.655, partial *η* ^2^ = 0.002), and no significant differences were found between intervention groups at any point (*F*[1,90] = 1.073, *p* = 0.303, partial *η* ^2^ = 0.012). The covariate ε4 status, on the other hand, had a significant main effect on IES‐R scores (*F*[1,90] = 46.376, *p* < 0.001, partial *η* ^2^ = 0.340), indicating that genetic risk influenced psychological distress. Additionally, the interaction between time × APOE ε4 status showed a trend toward significance (*F*[1,90] = 3.736, *p* = 0.056, partial *η* ^2^ = 0.040), suggesting that the decline in distress over time may have been moderated by genetic risk, although this effect did not reach statistical significance.

*χ* ^2^ Tests examined whether behavioral adaptations (yes/no) differed between the online and onsite intervention groups at 3 and 6 months. No significant differences were found between the two intervention groups at 3 (*χ* ^2^[1, *N* = 93] = 0.004, *p* = 0.951) or 6 months (*χ* ^2^[1, *N* = 93] = 0.088, *p* = 0.767).

### 3.3 MCI Compared to Cognitively Healthy Individuals

A mixed ANCOVA was conducted to examine changes in GCOS‐24 scores over time, comparing MCI vs. healthy participants while controlling for APOE4 status. Results showed a significant main effect of time (*F*[1,90] = 745.154, *p* < 0.001, partial *η* ^2^ = 0.892), indicating that empowerment significantly increased across all participants. There was also a significant time × diagnosis interaction (*F*[1,90] = 20.090, *p* < 0.001, partial *η* ^2^ = 0.182), suggesting that the rate of improvement differed between MCI and healthy groups. Additionally, the time × APOE ε4 interaction was significant (*F*[1,90] = 44.172, *p* < 0.001, partial *η* ^2^ = 0.329), meaning that genetic risk also influenced changes in empowerment over time. Lastly, a between‐subjects effect analysis showed that MCI participants had significantly lower overall GCOS‐24 scores than healthy individuals (*F*[1,90] = 78.949, *p* < 0.001, partial *η* ^2^ = 0.467), while APOE ε4 status alone did not have a significant effect (*F*[1,90] = 1.581, *p* = 0.212, partial *η* ^2^ = 0.017). A Bonferroni‐adjusted pairwise comparison confirmed this difference, showing that MCI participants had significantly lower GCOS‐24 scores than healthy individuals (mean difference = −16.627, SE = 1.871, *p* < 0.001, 95% CI = −20.345, −12.909), reinforcing the impact of cognitive status on empowerment levels.

Since normality and residual assumptions were clearly violated in the BDI scores, Mann–Whitney *U* tests were conducted to examine the effect of diagnosis (MCI vs. healthy) at each time point. The results indicated no significant differences in BDI levels between MCI and healthy participants at any measurement point: baseline (*U* = 1146.000, *p* = 0.582), 3 months (*U* = 1199.000, *p* = 0.335), and 6 months (*U* = 1145.500, *p* = 0.581). The same results were found for SAST scores, with no significant differences between MCI and healthy participants at any time point: baseline (*U* = 1010.500, *p* = 0.615), 3 months (*U* = 1006.000, *p* = 0.593), and 6 months (*U* = 1020.500, *p* = 0.671). Friedman tests also showed no significant changes over time in either BDI scores (*χ* ^2^(2) = 3.644, *p* = 0.162) or SAST scores (*χ* ^2^(2) = 4.414, *p* = 0.110), suggesting that levels of depression and anxiousness remained stable across all time points within both groups. For IES‐R, an ANCOVA showed that scores decreased significantly over time in both groups (*F*[1,90] = 31.467, *p* = 0.001, partial *η* ^2^ = 0.259), with no statistical difference between MCI and healthy people at any time point (Test‐between‐subjects; *F*[1,90] = 0.005, *p* = 0.944, partial *η* ^2^ = 0.000). Again, the controlled covariate APOE ε4 status had a significant main effect on IES‐R scores (*F*[1,90] = 46.413, *p* < 0.001, *η* ^2^ = 0.340). There was no difference in behavioral change between MCI and healthy participants at 3 (*χ* ^2^[1, *N* = 93] = 0.031, *p* = 0,860) and 6 months (*χ* ^2^[1, *N* = 93] = 0.457, *p* = 0.499). Table 3 summarizes the differences between groups.

**TABLE 3:** MCI/healthy participants: summary of between‐group differences.

Measure/domain	Between‐group difference	Statistical results
GCOS‐24 (empowerment)	Yes^a^	*F*(1,90) = 78.949, *p* < 0.001, *η* ^2^ = 0.467
IES‐R	No^b^	n.s.^d^
BDI	No	n.s.
SAST	No	n.s.
Behavioral adaptations (after 3 and 6 months)	No	n.s.
Risk recall (after 3 and 6 months)	Yes^c^	3 months: *χ* ^2^ = 8.40, *p* = 0.004, *Φ* = −0.30 6 months: *χ* ^2^ = 20.15, *p* < 0.001, Φ = −0.47

*Note:* Total *N* = 93.

Abbreviation: n.s. = not significant.

^a^ GCOS‐24: MCI participants had significantly lower GCOS‐24 scores compared to healthy individuals (*F*[1,90] = 78.949, *p* < 0.001, partial *η* ^2^ = 0.467).

^b^ For the IES‐R scores, the controlled covariate “ε4 status” was significant (*F*[1,90] = 46.413, *p* < 0.001, *η* ^2^ = 0.340).

^c^ Risk recall was significantly lower for MCI participants compared to healthy participants at 3 months (*χ* ^2^[1, *N* = 93] = 8.40, *p* = 0.004, *Φ* = −0.30) and 6 months (*χ* ^2^[1, *N* = 93] = 20.15, *p* < 0.001, *Φ* = −0.47). Diagnosis remained a significant predictor of Risk recall at 6 months even after adjusting for age and education (*p* = 0.032, Exp[*B*] = 0.089, 95% CI = 0.010–0.871).

^d^ Logistic regression was performed to examine whether diagnosis status (MCI vs. healthy) influences the ability to recall the genetic test results 3 and 6 months after testing (risk recall). The results confirmed that diagnosis is a highly significant predictor (OR = 0.215, 95% CI = 0.072–0.641) when no other predictors are included in the model. When adding education and age to the model, diagnosis becomes insignificant, meaning that the confounders mitigate the relationship between diagnosis and outcome (OR = 0.513, 95% CI = 0.138–1.916).

Risk recall was substantially lower among MCI participants (58%) compared to healthy participants (97.7%). Diagnosis remained a significant predictor in logistic regression (OR = 0.033, 95% CI = 0.006–0.361), indicating a 96.7% lower likelihood of recall in the MCI group. Even after adjusting for education and age, diagnosis remained significant (OR = 0.089, 95% CI = 0.010–0.871). Participants with an MCI diagnosis had 0.089 times the odds or 91.1% lower odds of successfully recalling risk compared to healthy participants (*p* = 0.032). Age was also a significant predictor, as older age reduced correct risk recall (OR = 0.888, 95% CI = 0.808–0.976). Additionally, for every 1‐year increase in age, the probability of successfully recalling risk decreased by 11.2%. Additionally, higher education increased the likelihood of recall (BOR = 5.798, 95% CI = 1.059–31.755), although the wide confidence interval to interpreting the magnitude of the effect of education with caution. Table 4 includes the main results of the logistic regression predicting risk recall accuracy after 6 months.

**TABLE 4:** Logistic regression predicting risk recall accuracy after 6 months.

Predictor	*p*	OR	95% CI
**Diagnosis (MCI vs. healthy)** ^a^	**0.032**	**0.089**	**[0.010, 0.817]**
Education^b^ (medium vs. low)	0.323	2.380	[0.427, 13.272]
**Education (high vs. low)**	**0.043**	**5.798**	**[1.059, 31.755]**
**Age**	0.**014**	**0.888**	**[0.808, 0.976]**
Constant	0.004	22952.97	—

*Note:* Total *N* = 93.

Abbreviations: 95% CI = 95% confidence interval; OR = odds ratio; *p* = statistical significance.

^a^ Diagnosis coded as MCI = 1, healthy = 0.

^b^ Reference category for education = low education (less than 6 years and primary school), medium education (high school diploma, 6–12 years of education), and high education (participants with university or equivalent higher education, more than 13 years).

### 3.4 Comparison of ε4‐Positive and ε4‐Negative Participants

A mixed ANCOVA examined GCOS‐24 changes over time between APOE ε4+ and APOE ε4− participants, controlling for diagnosis (MCI vs. healthy). GCOS‐24 scores significantly increased over time (*F*[1,90] = 630.926, *p* < 0.001, *η* ^2^ = 0.875), with a time × APOE ε4 interaction (*F*[1,90] = 44.172, *p* < 0.001, *η* ^2^ = 0.329), suggesting differences in the rate of improvement based on genetic risk. Diagnosis also interacted with time (*F*[1,90] = 20.090, *p* < 0.001, *η* ^2^ = 0.182), indicating an influence of cognitive status. Between‐group effects showed no significant main effect of APOE ε4 (*F*[1,90] = 1.581, *p* = 0.212, *η* ^2^ = 0.017), and pairwise comparisons confirmed no significant difference (*p* = 0.212). These results suggest that while empowerment improved over time, the rate of change varied by APOE ε4 status, but overall levels did not differ between genetic risk groups.

For BDI scores, nonparametric tests were conducted due to violations of normality. A Friedman test indicated no significant differences in BDI scores over time, *χ* ^2^(2) = 3.644, *p* = 0.162. Similarly, Mann–Whitney *U* tests showed no significant differences in BDI scores between APOE ε4+ and APOE ε4− participants at any time point: baseline (*U* = 1295.500, *p* = 0.095), 3 months (*U* = 1385.000, *p* = 0.687), and 6 months (*U* = 1425.500, *p* = 0.981). These findings suggest that BDI scores remained stable over time and did not differ based on APOE ε4 status. Similar results were found for SAST scores. Nonparametric tests indicated no significant differences between APOE ε4+ and APOE ε4− participants or over time. A Friedman test showed no significant change in SAST scores over time, *χ* ^2^(2) = 4.414, *p* = 0.110. Likewise, Mann–Whitney *U* tests confirmed no significant differences between genetic groups at baseline (*U* = 903.000, *p* = 0.168), 3 months (*U* = 940.500, *p* = 0.193), and 6 months (*U* = 1023.000, *p* = 0.744).

Additionally, participants who were carriers of the APOE ε4–gene consistently showed higher IES‐R scores compared to ε4‐negative (*F*[1,91] = 48.695, *p* < 0.001, partial *η* ^2^ = 0.349). There was a general decrease in IES‐R scores over time (*F*[1,91] = 147.334, *p* < 0.001, partial *η* ^2^ = 0.618), and this decrease was similar in both groups (*p* > 0.05, *η* ^2^ = 0.038).

The results of the *χ* ^2^ tests examining the association between genetic test results and behavioral change indicate a significant and strong association between APOE ε4 status and behavior change endorsement at both 3 months (*χ* ^2^[1, *N* = 93] = 32.594, *p* < 0.001, *Φ* = 0.592) and 6 months (*χ* ^2^[1, *N* = 93] = 36.471, *p* < 0.001, *Φ* = 0.626). APOE ε4–positive individuals were significantly more likely to endorse behavior change, as evidenced by the statistical significance and large effect sizes. At 3 months, 51.1% of APOE ε4–negative individuals reported no behavior change, while 48.9% reported a change. In contrast, 100% of APOE ε4–positive individuals endorsed behavior change. Since no APOE ε4–positive participant failed to change their behavior, logistic regression could not be conducted due to a lack of variance in the dependent variable. Table 5 summarizes the differences between the two groups of APOE ε4–positive and APOE ε4–negative.

**TABLE 5:** Summary of between‐group differences for APOE ε4–positive/APOE ε4–negative.

Measure/domain	Between‐group difference	Statistical results
GCOS‐24 (empowerment)	No^a^	n.s.
IES‐R	**Yes** ^**b**^	*F*[1,91] = 48.695, *p* < 0.001, partial *η* ^2^ = 0.349
BDI	No	n.s.
SAST	No	n.s.
Behavioral adaptations (after 3 and 6 months)	**Yes** ^**c**^	3 months: *χ* ^2^ = 32.59, *p* < 0.001, *Φ* = 0.592 6 months: *χ* ^2^ = 36.47, *p* < 0.001, *Φ* = 0.626
Risk recall (after 3 and 6 months)	No	n.s.

*Note:* Total *N* = 93.

Abbreviation: n.s. = not significant.

^a^ For GCOS‐24, the covariate “Diagnosis” was significant (*F*[1,90] = 12363.80, *p* < 0.001, partial *η* ^2^ = 0.467).

^b^ For IES‐R, participants with a positive genetic result showed significantly higher IES‐R scores compared to participants with a negative result (*F*[1,91] = 48.695, *p* < 0.001, partial *η* ^2^ = 0.349).

^c^ Behavioral adaptations differed significantly by APOE4 status at both time points: 3 months (*χ* ^2^ = 32.59, *p* < 0.001, *Φ* = 0.592) and 6 months (*χ* ^2^ = 36.47, *p* < 0.001, *Φ* = 0.626).

However, descriptive analysis was conducted to examine the specific behavioral changes adopted by participants, focusing on the most common strategies and their sustainability over 6 months. The most frequently reported behavior changes fell into the categories of “diet and medication” and “physical and cognitive exercise,” with 30 participants (32.3%) endorsing changes in both. An additional 11 participants (11.8%) reported changing only their diet and medication, while 12 participants (12.9%) reported adopting only physical and cognitive exercise. Seventeen participants (18.3%) reported more complex behavioral adaptations, which included combinations involving sleep, psychosocial strategies, or three or more simultaneous lifestyle domains. Meanwhile, 23 participants (24.7%) reported no behavioral changes.

Regarding the risk recall answers, the *χ* ^2^ tests did not indicate a significant association between gene status and risk recall at 3 (*χ* ^2^[1, *N* = 93] = 0.585, *p* = 0.444) and 6 months (*χ* ^2^[1, *N* = 93] = 0.030, *p* = 0.862).

### 3.5 Overall Changes Over Time

**FIGURE 3:**
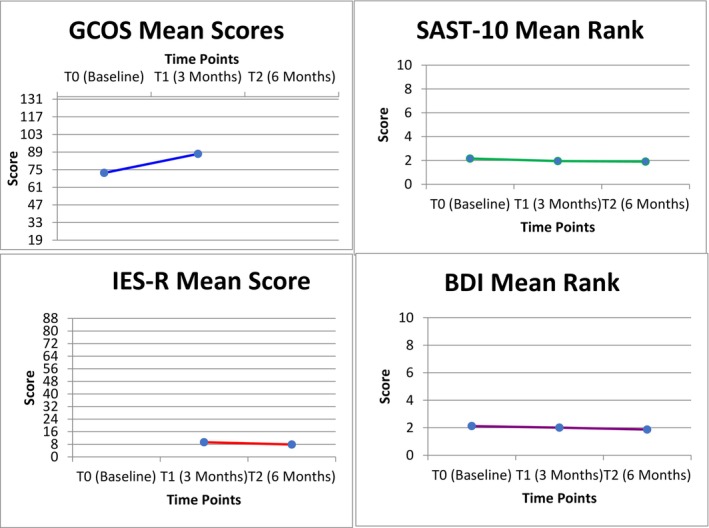
Overall changes in scales. *Note:* GCOS‐24 score range = *x*–*x*; IES‐R mean score = range *x*–*x*; BDI mean score range = *x*–*x*, SAST‐10 mean score range = *x*–*x*; GCOS‐24 and IES‐R were analyzed using paired‐samples *t* tests due to continuous, normally distributed scores. BDI and SAST scores, which violated normality assumptions, were analyzed using Friedman tests; therefore, mean ranks are presented.

The fifth research question explored whether the increase in empowerment scores (GCOS‐24), reductions in psychological distress (SAST, BDI, IES‐R), and behavioral changes were maintained at 3 and 6 months after the disclosure of genetic test results. The results are presented in Figure 3. There is a significant increase in the mean score of GCOS (baseline) (M = 72.44, SD = 10.669) and GCOS T1 (M = 87.55, SD = 14.389). The results (*t*[92] = −23.740, *p* < 0.001, *d* = −2.462) confirm that this difference is statistically significant and has a very large effect size. Similarly, there is a significant decrease in the mean score from IES‐R T1 (M = 9.31, SD = 2.532) to IES‐R T2 (M = 7.88, SD = 2.279). The results confirm that this difference is statistically significant and has a large effect size (*t*[92] = 12.036, *p* < 0.001, *d* = 1.248). After visually inspecting both scales, it is evident that the large effect sizes are not artifacts of major ceiling effects or distribution issues. While these effects may demonstrate high practical relevance, we must remain mindful of the limitations regarding the generalizability of Cohen *d*, as discussed by Lakens (2013), which underscores the importance of contextual factors in interpreting effect sizes [47].

Due to moderate violations of normality, the presence of outliers, and nonhomogeneous distributions in BDI and SAST scores—confirmed by both statistical tests and visual inspection—a nonparametric approach Friedman test was used. The results revealed no significant changes over time in both BDI scores (*χ* ^2^[2] = 3.644, *p* = 0.162) and SAST scores (*χ* ^2^[2] = 4.414, *p* = 0.110). We used a related sample McNemar's test to examine whether behavioral changes observed at 3 months were maintained at 6 months. The exact binomial test indicated no significant difference between time points (*p* = 0.500), suggesting that participants who adopted behavioral changes early on tended to sustain them over time. A majority (68 participants, 73.1%) who changed their behavior at 3 months remained consistent at 6 months, demonstrating a stable commitment to adaptation. Only two participants (2.2%) transitioned from no behavior change at 3 months to adopting change at 6 months, indicating minimal late adoption. Meanwhile, 23 participants (24.7%) who did not report behavior change at 3 months remained unchanged at 6 months.

## 4 Discussion

Overall, the results suggest that both online and face‐to‐face GC interventions are equally effective for disclosing the APOE genetic risk for AD to first‐degree relatives. Τhe participants did not show clinically significant depression or anxiety after the announcement of an increased risk for AD. In our study, it reduces concern about AD and causes behavioral changes, and these improvements continue over time. The same result was found among healthy individuals and those with MCI. However, comparing these groups revealed differences in risk recall scores, with MCI participants having substantially lower odds of recalling risk after 6 months and lower empowerment scores. When ε4‐positive people were compared with negative participants, ε4‐positive people were more likely to endorse behavior change and had higher IES‐R scores.

### 4.1 Online Compared to Onsite Intervention

Our results demonstrate that GCOS‐24 scores increased significantly over time across all participants, regardless of the type of intervention. These findings agree with the research conducted by Pestoff et al. (2022), in which a clinically important increase in empowerment was achieved on a group level 2 weeks after tele‐GC [4]. To our knowledge, this is the first study to measure the effects on participants' empowerment after online GC compared to onsite GC. Τhe first study by Pestoff et al. (2022) measured the impact of tele‐GC using the GCOS‐24, but there was a lack of including a comparison with an onsite intervention [4]. The findings of this study were concordant with many research studies focused on face‐to‐face GC interventions and underlined a significant improvement in empowerment after the intervention [48, 49, 50]. For instance, in one study, 1479 participants in GC sessions completed the GCOS at three time points, and half of them showed clinically relevant improvement in empowerment [48].

Additionally, regarding the comparison of online and onsite groups, no significant differences were found in BDI, SAST, or IES‐R scores. In addition, there was no significant difference in behavioral change between the two intervention groups, meaning that they showed similar levels of behavioral change at 3 and 6 months from baseline. It is also notable that test‐related distress decreased significantly over time in both groups. This evidence supports the equal effectiveness of videoconferencing to in‐person delivery of clinical genetics services. These results are consistent with results from prior trials that demonstrated that remote GC is non‐inferior to onsite [2, 3, 51, 52, 53]. These findings are encouraging, given that many genetic service providers are already disclosing test results via remote means. It is crucial to emphasize that there are not many studies, until now, which focus on the psychological outcomes of participants of online GC sessions. Most studies on tele‐genetics investigated the high satisfaction of participants, as well as the benefits and barriers of remote GC [2, 52, 53, 54]. The results of these studies demonstrate a continuing demand for remote delivery of GC, which underlined the benefits of flexibility and cost‐ and time‐saving.

A systematic review reported that anxiety and depression levels decreased over time in participants who received tele‐genetics counseling [2]. Another more recent systematic review comparing videoconferencing with face‐to‐face sessions for the delivery of clinical genetics services highlighted that patients who received tele‐GC had immediate outcomes at least equivalent to those of patients who received face‐to‐face counseling—in terms of satisfaction, knowledge gained, and psychosocial outcomes (depression and anxiety) [3]. Additionally, similar responses have been observed in a study by Christensen et al. (2018) comparing in‐person to telephone disclosure of genetic risk information about ad [55]. Telephone disclosure was safe and did not increase psychological risks such as anxiety, depression, and distress. In a comparison of an online GC group with a control group, Otten et al. (2016) found that online participants did not differ from the control group in decreased anxiety and increased sense of control [51]. My interpretation of these results is that this may indicate participants who choose online counseling are generally less anxious than those who prefer face‐to‐face counseling. As most studies agree, tele‐GC has been proven to have successful implementation, preliminary efficacy, and acceptance by participants compared to face‐to‐face GC, which could also explain our results [4]. Another explanation for our findings could be that new technologies are more acceptable and user‐friendly after the COVID‐19 pandemic, adding significant benefits to online GC sessions. In Greece, for instance, GAADRD continued to offer non‐pharmacological interventions during the COVID‐19 pandemic using online delivery methods, making videoconferencing accessible to those with MCI. It is also important to emphasize that we did not encounter any challenges in providing GC virtually, nor did we experience difficulties with technology. In cases of minor difficulties, the genetic counselor provided specific instructions to participants or rescheduled the session to a time when a companion could be presentor provided specific instructions to participants, or the session was rescheduled to a time when a companion could be present.

### 4.2 MCI Compared to Cognitively Healthy Individuals

Many studies have demonstrated that genetic risk assessments for AD based on APOE genotyping do not increase risks for psychological harm when provided in clinical settings to asymptomatic individuals [13, 16, 18, 19, 55, 56]. However, until now, few studies have included individuals with MCI who are at higher risk for AD in the near future. Therefore, questions remain regarding whether genetic risk disclosure is safe for individuals experiencing memory problems who may progress to AD dementia shortly [11]. MCI introduces unique complexities to the communication process, as these individuals tend to have difficulty processing often abstract and complex information [57]. Nevertheless, the results of this study indicate no differences in depression, anxiety, risk‐related distress, and behavioral adaptations between MCI and healthy individuals at any time point. Similar responses have been observed in other studies, which proved that providing genetic information to individuals with MCI about imminent risk for AD can offer psychological benefits and does not increase the risks of anxiety or depression [11]. Nonetheless, they found that MCI participants' distress responses to risk disclosure appeared to be greater than in prior studies of healthy, at‐risk adults. Another study examined the relationship between subjective cognitive decline (SCD) participants and the impact of APOE disclosure on psychological and behavioral outcomes and found that the presence of SCD did not exacerbate the AD risk‐related distress following the announcement that one was a ε4 carrier [58].

One measure commonly used to assess the efficacy of risk communication is risk recall. When participants in disclosure sessions do not accurately recall and interpret the genetic risk information received, it may lead to inappropriate decision‐making [41]. Our results demonstrate that healthy participants were more likely to remember the risk than those with MCI, which was expected due to their mild memory issues. Understanding complex information such as genetic risk is unfamiliar and challenging for many individuals with disorders, as well as for older, less literate, and medically complex adults, especially those with cognitive deficits [57, 59]. Similar responses were observed in the REVEAL study, which assessed how well healthy individuals could remember their AD risk test results at various points after disclosure. It found that 31% could not recall their APOE genotype after 6 weeks [42]. Researchers proposed that characteristics such as demographic factors, psychological states, and numeracy influenced participants' ability to remember the personal genetic risk information they received [42]. Additionally, in a related study by Besser et al. (2015), 26% of individuals were unable to recall their APOE genotype [41]. They noted that genetic risk information may involve different cognitive demands or be affected by various factors during recall. It is also crucial to emphasize that studies focusing on SCD found it difficult for the group with SCD to recall details of their APOE status compared to healthy participants [58].

Our results also demonstrate that higher education increased the likelihood of risk recall, while older age reduced it, further confirming previous research that has found educational levels and age are distinct determinants of recall. These factors are linked with the demographic characteristics of MCI people as they are older and have a lower educational level and finally face difficulties in risk recall. Similarly, in the study by Besser et al. (2015), the correct recall of the number of APOE risk‐increasing alleles was independently associated with higher education and greater numeracy, and correct recall of the lifetime risk estimate was independently associated only with younger age [41].

The outcomes also reveal that individuals with MCI have a lower empowerment score, which correlates with their cognitive deficits. Empowerment is defined as a combination of cognitive, decisional, and behavioral control, emotional regulation, and hope. The GCOS‐24 is structured around these dimensions and includes items related to the cognitive dimension. Cognitive control involves understanding the condition and its personal and familial impacts, considering the potential risks for oneself and one's loved ones, and being informed about healthcare and other resources. Additionally, empowered participants in GC sessions are more likely to see themselves as primary leaders in managing their health and to follow the recommendations and advice provided during appointments [50]. Therefore, another possible interpretation is that because people with MCI already experience cognitive deficits and are closer to AD, they may feel less able to control their health conditions.

### 4.3 Comparison of ε4‐Positive and ε4‐Negative Participants

As described in other studies, we also found in this trial that ε4‐positive subjects showed no more symptoms of general anxiety or depression than ε4‐negative subjects [13, 18, 19, 55, 60]. These findings are consistent with results from prior trials that demonstrated the safety of disclosing genetic risk information about AD when provided to populations in a well‐designed education and disclosure protocol [13, 18, 19, 55, 60] and that showed psychological benefits and more positive feelings for participants who learned that they were ε4‐negative [15]. Also, it was highlighted that MCI participants were expecting bad news, given their mild memory deficits and positive psychological outcomes likely accrued by the negative genetic result [11].

The outcomes also reveal that both groups faced a general decrease in test‐specific distress scores over time, but ε4‐positive participants showed higher scores. This result is in line with the study by Christensen et al. (2020) in which ε4 carriers showed greater distress at all follow‐ups that were clinically insignificant but statistically important [11]. In another study, the level of risk‐related distress stayed subclinical in both ε4‐positive and ε4‐negative individuals, but ε4‐positive individuals perceived a greater risk for developing AD, and these scores held over the 6‐month follow‐up time point [56]. However, perceived risk in the ε4‐negative group reduced significantly and stabilized over the 6‐month follow‐up, and ε4‐positive people were more likely than ε4‐negative to remember their APOE genotype after the period of 6 months.

Similar responses have been observed in other studies, in which ε4 carriers were more nonverbally negative, indicating that they were experiencing and displaying obvious distress and anxiety when receiving their results [48, 57]. Further supporting this rationale, results on SCD participants demonstrated that receiving an ε4‐positive result caused a modest, temporary impact on mood and risk‐related distress, but the scores were subclinical and decreased to baseline after the 6‐week follow‐up [58]. These findings highlight the increased risk for distress in disclosing ad risk information to individuals who are ε4 carriers and the need for well‐designed education, communication, and follow‐up protocols when providing risk information to individuals who received ε4‐positive outcomes.

The outcomes also reveal that there were no differences between ε4‐positive and ε4‐negative participants regarding the scores of GCOS. However, according to Largent et al. (2021), ε4 noncarriers characterized their positive emotional reactions as empowerment. They explained that knowing their genetic results enabled them to “prepare ahead of time… prepare for my later life” and to “make plans based upon the probabilities that I would develop AD” [15]. One possible interpretation of our results regarding the similar scores in GCOS of ε4 carriers and noncarriers could be that all post‐GC surveys were performed 3 months after the genetic test result disclosure, in contrast with other studies, which measure GCOS immediately after disclosing the results. Therefore, empowerment scores could be influenced by genetic testing outcomes.

There were also no differences between ε4‐positive and ε4‐negative participants regarding the risk recall. These data are partially in contrast with what was observed in other studies, for instance, that ε4 carriers were more likely to correctly recall their genotype than ε4 noncarriers [42, 55, 56]. Individuals who were ε4‐positive likely saw a greater need to remember this information, given their increased risk status [55].

According to our results, people who are carriers of the ε4 allele are much more likely to endorse behavior change. This result is similar to other studies [15, 19, 60, 61, 62, 63, 64], which reveal that ε4‐positive people adopted new health behaviors at higher rates than ε4‐negative. In addition, in one study, 50% of ε4 homozygotes reported changes in health behaviors, while just under 30% of heterozygotes and noncarriers reported similar changes [15]. One hypothesis is that ε4 carriers approach healthier lifestyle changes with the idea of reducing their higher risk. Also, these people had higher scores on the distress scale over time, which can also explain their tendency for behavioral change. This explanation is consistent with the findings of Oliveri et al.(2022), who emphasized that as anxiety can act as a mechanism for lifestyle and health behavior changes, in their study, those who reported actually changing their lifestyle at 6 months had significantly higher levels of anxiety than those clients who made no changes [65]. The same study also demonstrated that participants who had received a negative result also preferred to adopt a healthier lifestyle after receiving the genetic test results. This result is in line with the findings of our study, as 48.9% of ε4‐negative individuals reported behavior change.

Our data are partially in contrast with what was observed in other studies, as no changes in protective health behaviors, especially for ε4‐positive participants, were declared [66, 67, 68]. Nielsen et al. (2017) investigated the effects of direct‐to‐consumer personal genomic testing on health behaviors and found that the experience of testing was associated with modest, mostly positive behavioral changes, but this association was independent of genetic results [66]. Also, in another study, no differences were observed in MCI people's ad prevention behaviors at 6 months, except for medication use; however, this was higher among participants who were ε4‐negative compared to participants who did not receive genotyping [55].

It is also crucial to emphasize that most of the above studies underlined the need for education about the role of lifestyle factors in AD risk and provided guidelines on making risk‐lowering lifestyle modifications as an intervention approach that leads to positive change. Our study protocol includes educational sessions with printed materials about the role of risk factors for AD, their cumulative impact, and risk‐modifying behaviors, which can explain the tendency for behavioral changes in our participants. Most studies underlined the need to give participants standardized education about risk‐modifying behaviors, present some form of actionable behavior change, and also explicitly inform participants that risk‐modifying behaviors have not been proven to affect disease progression [16, 20, 61]. The influence of the type of education that will be provided to this population and the mode of delivery needs further investigation.

Studies also presented the behavioral changes that participants followed after disclosure of their genetic information. According to the results of the present study, the most common behavior changes occurred in the “diet and medication” and “physical and cognitive exercise” categories. These findings align with other studies, which revealed that the most common changes were made in diet and exercise. One possible explanation for our results is that many studies on the Mediterranean diet and natural products have been conducted in Greece and are linked to a potential decrease in AD risk. These results have been communicated to the general public. Additionally, another explanation for the high scores in the “physical and cognitive exercise” categories is that some participants came from GAADRD and had prior experience with cognitive activities and their impact on improving cognitive dimensions. Similarly, Chao et al. (2008) found that ε4 carriers are more likely to have changed their medications or vitamins, even though they explicitly informed participants that risk‐modifying behaviors have not been proven to affect disease progression found that ε4 carriers are more likely to have changed their medications or vitamins, even though they explicitly informed participants that risk‐modifying behaviors have not been proven to effect disease progression [63].

Additionally, in other studies, ε4‐positive individuals were more likely than ε4‐negative individuals to report changes specifically related to mental activities (38% vs. 19%, *p* < 0.001) and diets (21% vs. 12%, *p* = 0.016) 6 weeks post‐disclosure [19]. In another study, ε4 carrier status was associated with physical activity but not with dietary habit scores [16]. Another analysis relating to the actual long‐term behavioral changes adopted by this sample following genetic testing showed that a healthier diet and a generalized sense of “awareness and attention” toward their health were the most frequently mentioned aspects. However, physical activity and preventive medical screening remained among the initial intentions of participants and were not confirmed as effective lifestyle changes adopted after 6 or 12 months [65]. Numerous other studies described behavioral changes resulting from genetic disclosure, emphasizing that participants improved their diets, exercised regularly, and, for some, used supplements. Although they do not know if these changes will prove effective in warding off AD, they feel healthier and mentally sharper than they would have been had they not undergone testing [61].

### 4.4 Overall Changes Over Time

According to the results of the present study, there is a significant increase in the mean score of GCOS over time. GC has been found to provide patients with a better knowledge of the disease and risk‐reducing measures, which were subsequently reported to empower participants in their decision‐making regarding genetic testing [49]. According to our results, 75.3% of the participants made behavioral changes, which could also be related to the increased empowerment scores. It is crucial to emphasize that the current results revealed that empowerment scores significantly increased after GC by 15.11 points (pre‐GC:72.44; post‐GC: 87.55), which is higher than the MCID for the GCOS‐24 of 10.3 points reported by Thomas and McAllister (2019) in the British population [32].

This study confirms that empowerment, as measured by the Greek GCOS‐19, could be considered a valuable outcome in GC. The Greek GCOS‐19 appears capable of measuring the goals of GC and changes in empowerment over time. New large outcome studies and more qualitative research are needed to define what might be considered normal and desirable effect sizes on GC outcomes.

It has also been proven that using PROMs in GC, like the GCOS, for monitoring the psychological status of participants, facilitates the discussion of their psychosocial problems and reduces their distress levels [69]. These results are supported by our data, which showed that the effect of time after the receipt of genetic test results reduced distress scores over time.

The finding that participants maintained their behavioral changes both at 3 and 6 months is consistent with results from prior trials [16, 63]. More specifically, Chao et al. (2008) demonstrated that ε4 carriers endorse health behavior change 12 months after disclosure [63]. Similarly, one study also confirmed long‐term positive effects on the participants' behavior and their health 6.5 years after the ε4 genetic risk announcement, indicating the potential for permanent health improvements [62]. They discovered that, during the 5.5‐year period between two different interventions, positive lifestyle changes had been observed, particularly by the ε4 carriers of the former control group, who were informed about their risk genotype only at the end of the first intervention.

## 5 Limitations

The present study has several limitations. First, it is important to highlight that our study excluded individuals with very severe anxiety and depression. Additionally, the volunteers who participated were predominantly female, well educated, younger, and positively inclined toward genetic testing. Consequently, individuals who might be less motivated to learn these results, who had higher levels of baseline distress, or who were older or less well educated might not exhibit the same outcomes. As a result, the generalizability of these findings may be limited. Future studies should target a group with greater diversity, including different genders, various age groups, and individuals with a range of educational backgrounds. The generally higher education levels among participants may also correlate with better preparedness to cope with higher‐risk results than the population at large.

Second, it is important to note that although our study required MCI participants to enroll and attend sessions with a companion (predominantly spouses or adult children) to ensure their safety, we did not explore the impact and role of visit companions in our results.

A further limitation of the use of GCOS‐24 in this study is that the Greek translation of GCOS‐24 was not evaluated for readability and interpretability through cognitive interviews with Greek individuals, as the other translations and adaptations of GCOS‐24 were [70, 71].

Another limitation is that the health behavior questions were nonspecific, and we measured responses to questions about changes in health behavior. Further studies are needed to focus on directly measuring the changes themselves [63].

Our study did not assess potential benefits or barriers of online GC such as satisfaction of participants, increasing access to testing, decreasing costs to patients, flexibility, and timesaving. Such analyses may be important for the Greek population, and future studies can address this issue.

Also, for individuals receiving genetic risk results for other common complex conditions such as diabetes or heart disease, a different set of outcomes (involving appropriate interpretation and subsequent behaviors) will likely be more important than anxiety, depression, and distress, and our study does not address this.

Additionally, this research was carried out in a single center. Counseling was provided by a single genetic counselor and thus, empowerment scores were likely impacted by the counselor's ability to target these specific topics via utilization of the GCOS in the session. This study design increases the standardization of approach and uniformity of counseling but may limit the generalizability of findings. Therefore, these outcome data may not accurately reflect what occurs in GC sessions, as these interventions may not be utilized in the same way by other genetic counselors.

Finally, another important piece of further research could be a large‐scale study and the implementation of GC across multiple countries or regions, which would provide a more comprehensive understanding of the cultural, socioeconomic, and healthcare factors that influence family members and patients. One of the biggest obstacles researchers face when validating an instrument is the difficulty of obtaining samples from different regions [72].

In conclusion, further research is needed to determine how best to communicate different types of genetic risk information to patients, particularly those with cognitive deficits, levels of education, and older age.

## Author Contributions

Conceptualization: M.M., I.A.A., and D.M.; writing – original draft: M.M.; writing – review and editing: M.M., I.A.A., D.M., L.F., T.T., V.K., and M.T.; data curation: M.M., I.A.A., and MT; methodology: M.M., D.M., and M.T.; formal analysis: M.M. and I.A.A.; investigation: M.M. and M.T.; visualization: M.M. and M.T.; supervision: D.M. and M.T.

## Ethics Statement

The study was conducted according to the guidelines of the Declaration of Helsinki and approved by the Research Ethics Committee of the School of Medicine of Aristotle University of Thessaloniki (Committee Approved Meeting Number: 1/19.10.2021) and also the Scientific and Ethics Committee of the Greek Association of Alzheimer's Disease and Related Disorders (Committee Approved Meeting Number: 91/07‐09‐2023).

## Consent

Informed consent was obtained from all subjects involved in the study.

## Conflicts of Interest

The authors declare no conflicts of interest.

## Data Availability

The data supporting the findings of this study are available on request from the corresponding author. The data are not publicly available due to privacy or ethical restrictions.
